# Supplementary figures and images for: Interaction between Sulfate and Selenate in Tetraploid Wheat (Triticum turgidum L.) Genotypes
Source: Int J Mol Sci. 2023 Mar 13;24(6):5443. doi: 10.3390/ijms24065443 (PMC10055959; doi:10.3390/ijms24065443)

Figure S1

S0

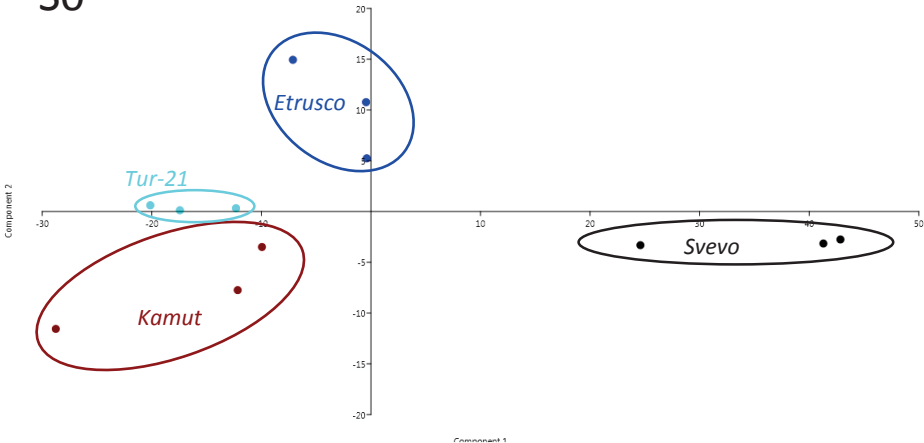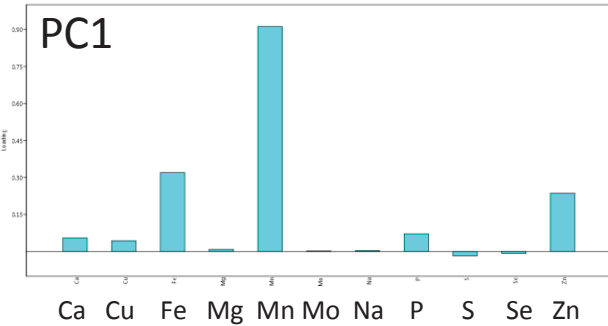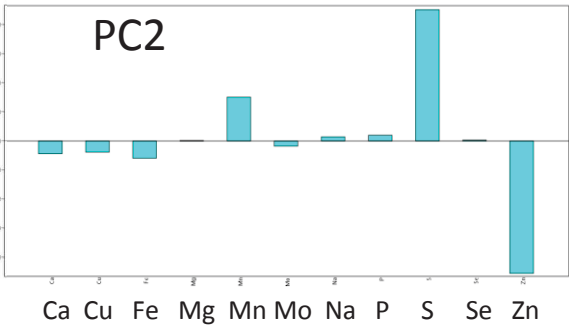

L0

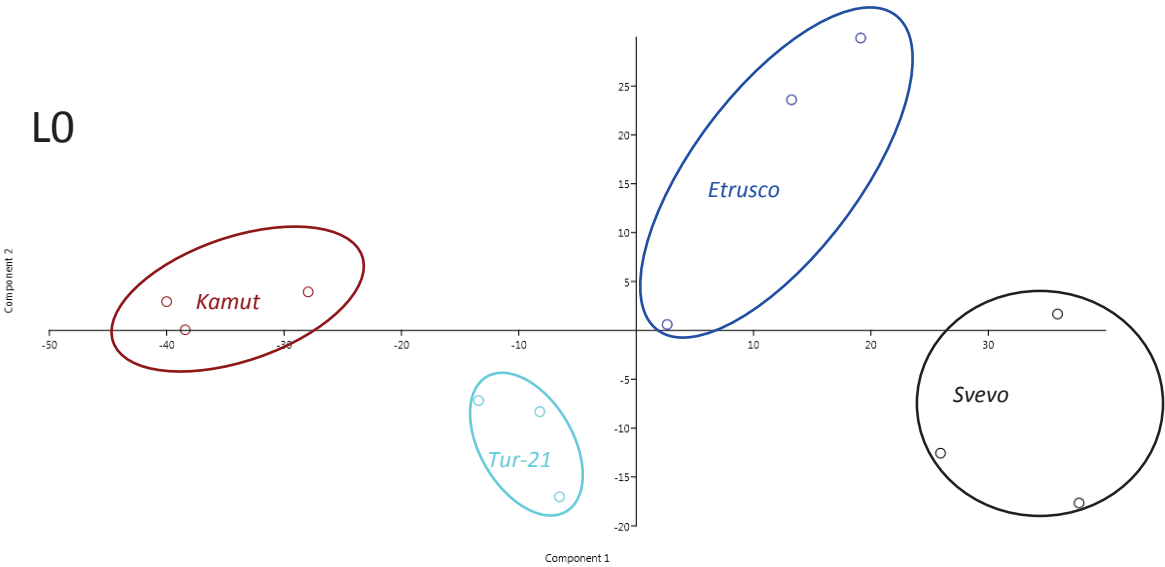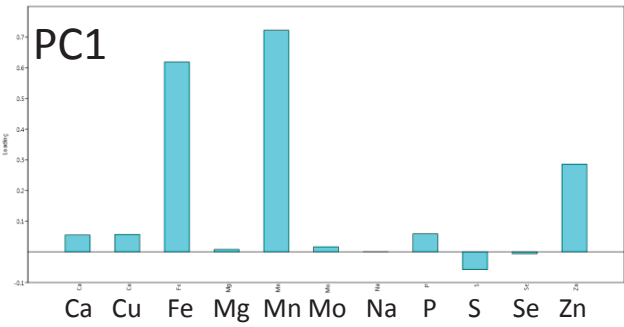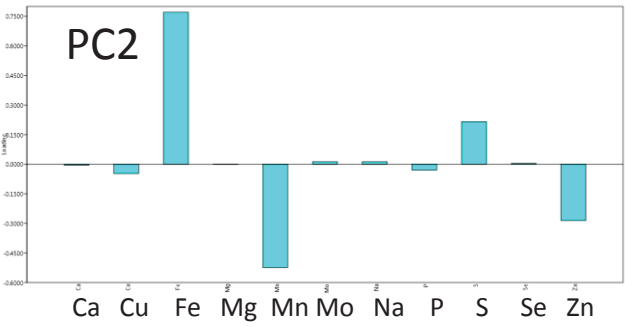

# S10

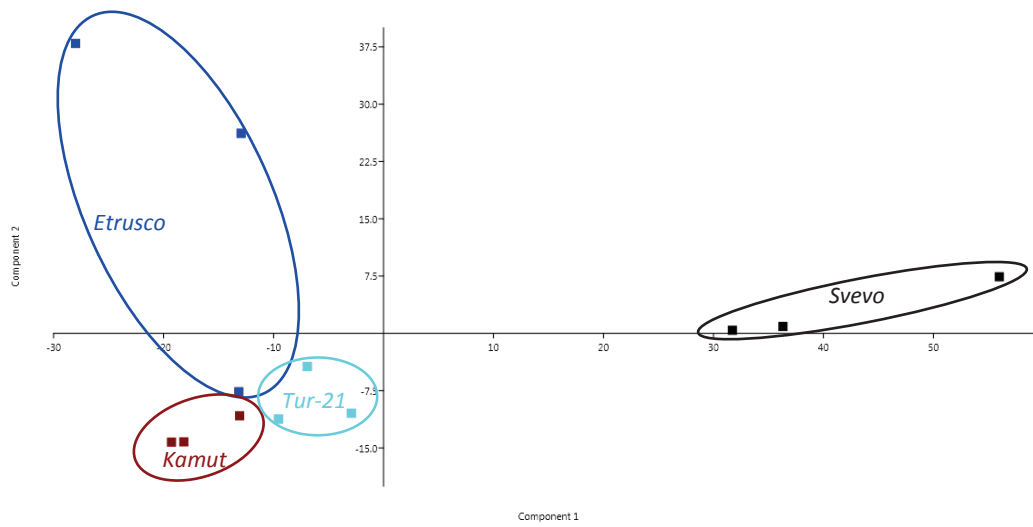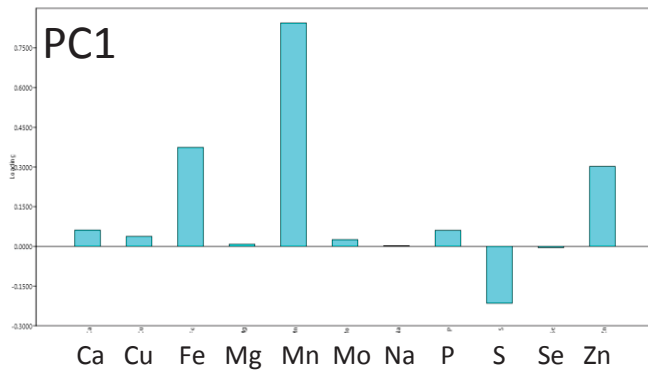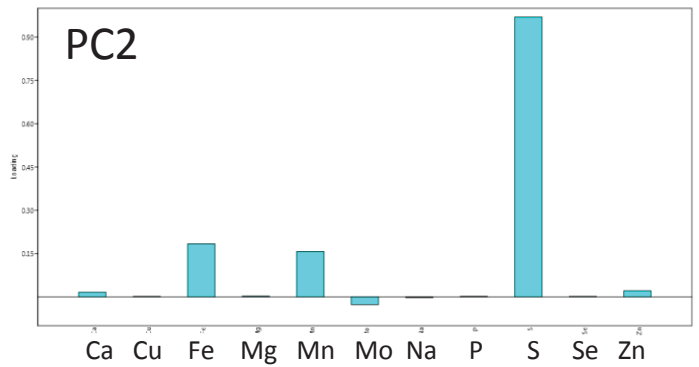

# L10

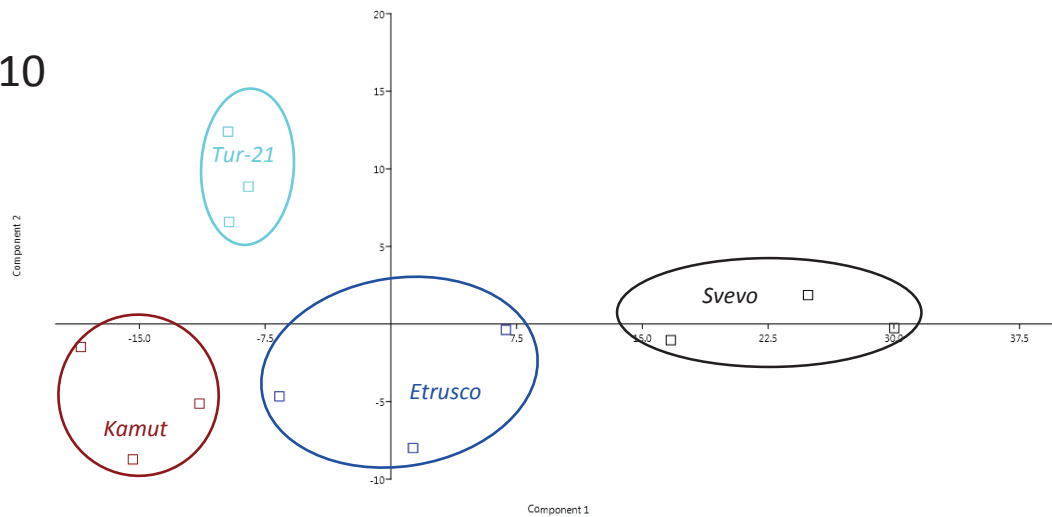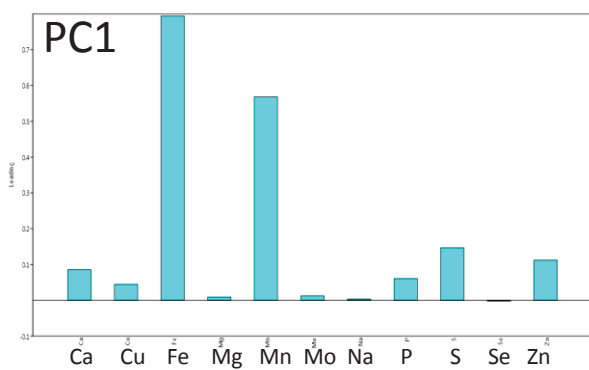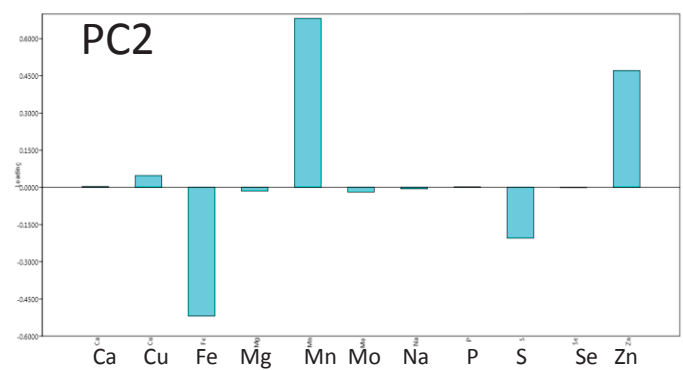

S50

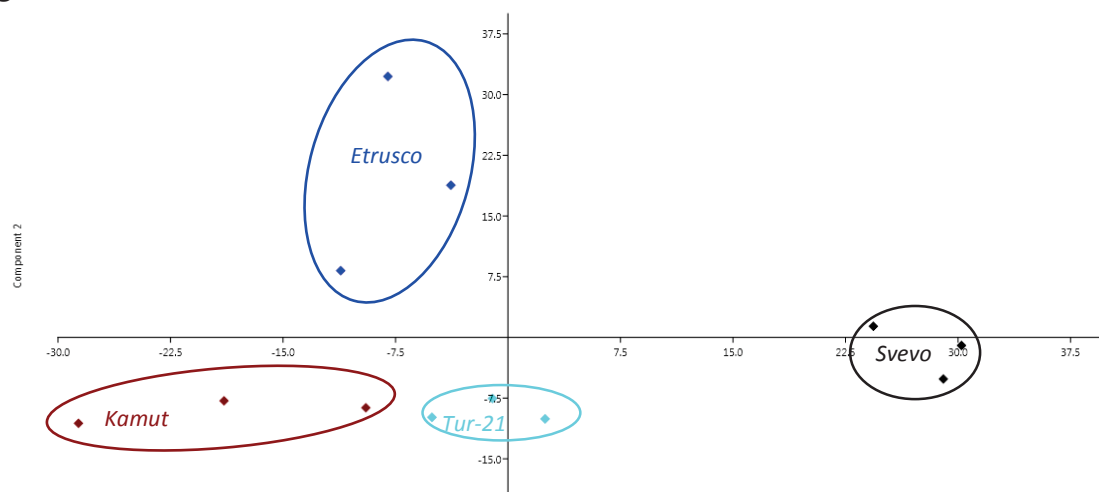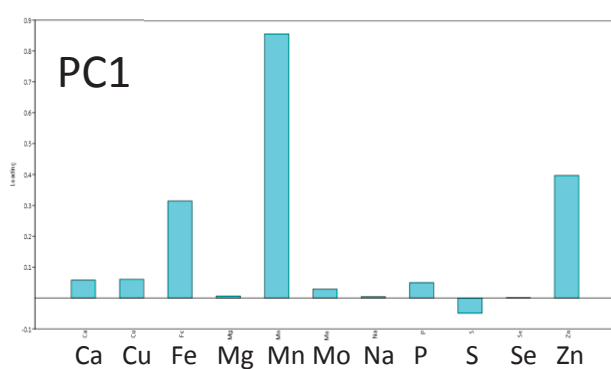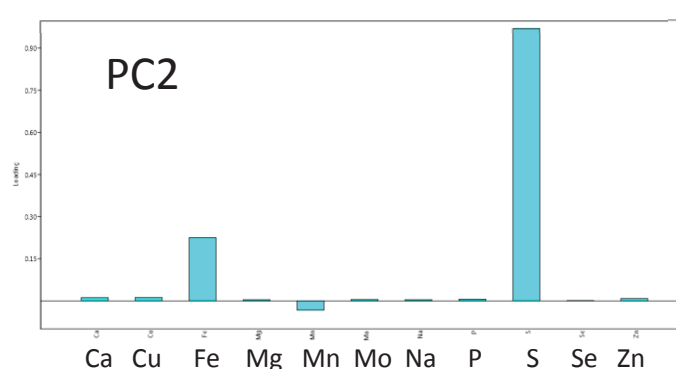

L50

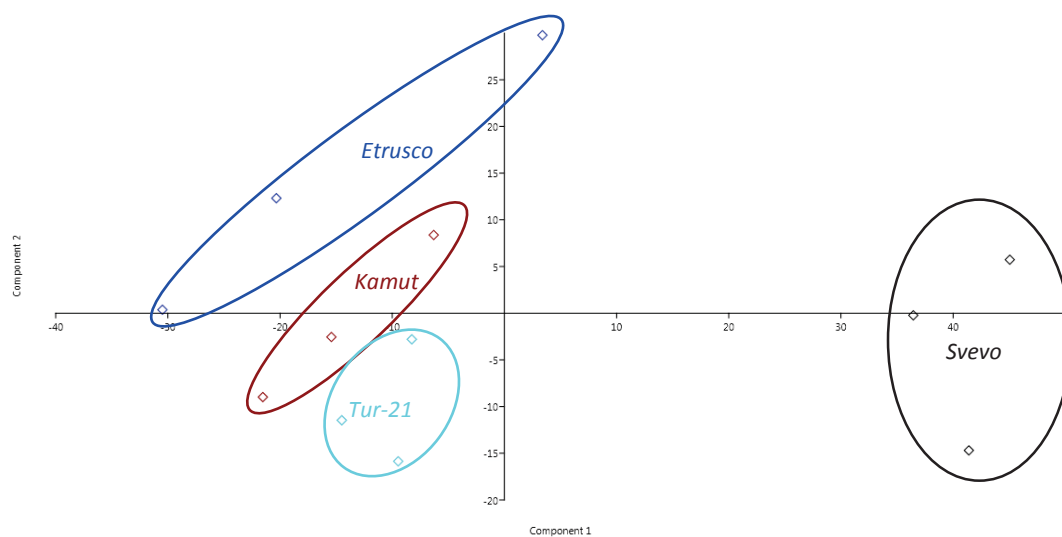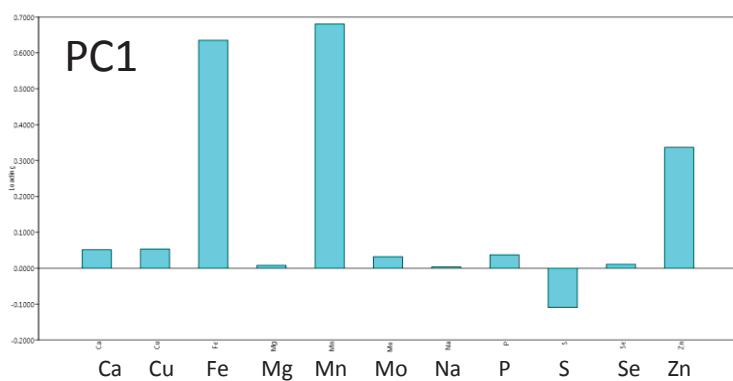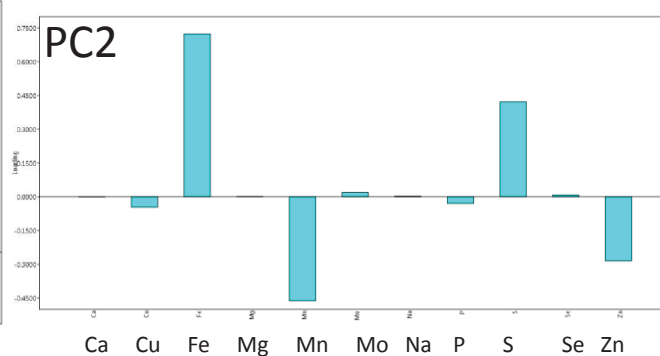

Supplement: Supplementary file 1 [file ijms-24-05443-s001.zip › ijms-2253630-supplementary.pdf]
